# Poster Session II - A294 DEPLETION OF COLONIC HNF4A ACCENTUATES *CITROBACTER RODENTIUM* INFECTION SEVERITY IN FEMALE MICE

**DOI:** 10.1093/jcag/gwaf042.294

**Published:** 2026-02-13

**Authors:** L M Leon Chirino, D Pupo Gomez, G Marrero Cofino, A de Castro, C Jones, A Menendez, L Fortier, F Boudreau

**Affiliations:** Immunology and Cell Biology, Universite de Sherbrooke Faculte de Medecine et des Sciences de la Sante, Sherbrooke, QC, Canada; Immunology and Cell Biology, Universite de Sherbrooke Faculte de Medecine et des Sciences de la Sante, Sherbrooke, QC, Canada; Immunology and Cell Biology, Universite de Sherbrooke Faculte de Medecine et des Sciences de la Sante, Sherbrooke, QC, Canada; Immunology and Cell Biology, Universite de Sherbrooke Faculte de Medecine et des Sciences de la Sante, Sherbrooke, QC, Canada; Immunology and Cell Biology, Universite de Sherbrooke Faculte de Medecine et des Sciences de la Sante, Sherbrooke, QC, Canada; Immunology and Cell Biology, Universite de Sherbrooke Faculte de Medecine et des Sciences de la Sante, Sherbrooke, QC, Canada; Immunology and Cell Biology, Universite de Sherbrooke Faculte de Medecine et des Sciences de la Sante, Sherbrooke, QC, Canada; Immunology and Cell Biology, Universite de Sherbrooke Faculte de Medecine et des Sciences de la Sante, Sherbrooke, QC, Canada

## Abstract

**Background:**

The transcription factor hepatocyte nuclear factor 4 alpha (HNF4A), an epithelial specific transcriptional regulator, is significantly decreased in inflammatory bowel diseases. The *Citrobacter rodentium* infection model in mice recapitulates human intestinal diseases associated with enteropathogenic *E. coli*. It is currently unclear whether the absence of HNF4A in the gut mucosa impacts the severity of *C. rodentium* infection-related intestinal diseases.

**Aims:**

Herein, we aimed to determine whether the loss of HNF4A in intestinal epithelial cells (IEC) renders the intestinal epithelium more susceptible to *C. rodentium* infection, thereby altering the gut microbiota composition and the function of goblet cells (GCs) and mucus layers, as preconditions leading to the onset of severe colitis.

**Methods:**

Control and *Hnf4a*^*ΔIEC-ind*^ mice of both sexes were challenged with *C. rodentium* and then monitored daily for body weight and fecal *C. rodentium* counts until bacterial clearance was achieved. Gut microbiota composition of cecum and colon fecal samples will be defined by 16S rDNA sequencing. The integrity of the GC and mucus layers upon *C. rodentium* infection will be evaluated using immunohistochemistry, immunofluorescence, immunoblotting, and gene expression analyses.

**Results:**

*Hnf4a* ^*ΔIEC-ind*^ female mice weighed significantly less than the controls from day 9 post-infection until the moment they cleared the bacteria. The course of the infection was erratic in the control group, whereas the female mutants exhibited a rapid and consistent infection, characterized by much higher levels of bacteria in their feces. Surprisingly, *Hnf4a*^*ΔIEC-ind*^ male mice did not differ significantly from the controls under these conditions. Preliminary observations suggested that the colonic base of crypts in GC was rapidly depleted in infected *Hnf4a*^*ΔIEC-ind*^ mice. Since mucin-degrading bacteria such as *Akkermansia muciniphila* were reported to be increased in *Hnf4a*^*ΔIEC*^ mice in the past, we expect to find signs of dysbiosis in *Hnf4a*^*ΔIEC-ind*^ mice associated with GC and mucus layer defects.

**Conclusions:**

These results support the notion that HNF4A function in IEC is essential for preserving epithelial defenses through the control of gut microbiota composition and the prevention of GC and mucus layer dysfunction, particularly in female mice. As future directions, we will measure the impact of the microbiota on GC and other IEC types in the context of colon organoid-derived monolayers in Transwells.

**Funding Agencies:**

CIHR

